# Redirecting Care: Compassionate Management of the Sick or Preterm Neonate at the End of Life

**DOI:** 10.3390/children9030344

**Published:** 2022-03-02

**Authors:** John Wyatt, Richard Hain

**Affiliations:** 1 University College London, London WC1E 6BT, UK; 2 Paediatric Palliative Medicine, Cardiff and Vale University Health Board, Clinical Ethics, Swansea University, Swansea SA2 8PP, UK; richard.hain@southwales.ac.uk

**Keywords:** neonates, palliative care, Ethics, symptom control

## Abstract

The primary moral commitment of medical care has traditionally been based on a belief in the intrinsic value and significance of human life and a desire to protect the most vulnerable from harm. In this respect, the care of newborn infants who are at the border of viability is no different. Despite the intrinsic value of the life of every newborn, all agree that there is no moral duty of doctors to provide every possible treatment where the prognosis is hopeless. Instead, every action and treatment should be orientated towards the best interests of the individual child and towards the minimisation of serious harm. Decisions about the withholding or withdrawal of life-supportive treatment should be made collaboratively between professionals and parents, with discussion starting prior to delivery wherever possible. The goals of neonatal palliative care are to prevent or minimise pain and distressing symptoms and to maximise the opportunity for private, loving interaction between the dying baby and his or her parents and the wider family. Physical contact, gentle stroking, cuddles and tender loving care are of central importance for the dying baby. At the same time, we must provide psychological support for parents and family as they go through the profound and painful life experience of accompanying their baby to death. To enable a baby to die well, pain-free and in the arms of loving parents and carers is not a failure but a triumph of neonatal care.

## 1. Introduction

The ideas of ‘intensive’ and ‘palliative’ care can seem diametrically opposed. The objective of intensive care is cure. Intensive care can restore a majority of babies to normality; even where that is not possible, it has the potential to enable a child to live for many years or decades. Palliative care, in contrast, starts with an acknowledgement that such long-term life is no longer possible. It is understandable that for healthcare professionals caring for neonates, redirecting from intensive to palliative care can feel like concession to hopelessness and perhaps even an admission of failure.

The aim of this chapter is to show that seen from an ethical perspective, the goals of intensive care and palliative care are not as remote from one another as they might appear. Although the expected outcomes are different, intensive care and palliative care are underpinned by the same compassion for a baby and her family and by the same commitment to improve the quality of their existence, up to (and including) the moment of the baby’s death.

## 2. Ethical Frameworks

The primary moral commitment of medical care has traditionally been based on a belief in the intrinsic value and significance of human life. In this respect, the practice of neonatology is no different from that of any other branch of medicine. We start with an implicit commitment to preserve and protect the life, health and well-being of the newborn under our care.

However, a belief in the intrinsic value of every human life does not translate into a moral duty of doctors to provide every possible treatment in every possible clinical circumstance. Instead, there is a general agreement that every medical intervention and practice must be orientated towards the best interests of the individual child.

It is not possible to consider the legal aspects of medical decision making in any detail in this article. Readers are drawn from many different legislatures, each with its own set of laws on medical decision making in children. In some countries, for example, doctors are legally obligated to act in accordance with parents’ preferences, whereas in others, it is parents who are expected to agree. Hence, it would be impractical to address the whole range of legislation meaningfully. It is, however, possible to identify ethical principles that are common between many different cultures. Even where it is not *legally* required, for example, it is reasonable to suggest that in all cultures, there is a *moral* imperative for doctors to respect parents’ views and to offer to engage them in dialogue.

In the UK, it is the concept of best interests that underpins child and family legislation and has been the basis for high-profile legal judgments in individual cases [1,2,3]. The concept of ‘best interests’ is intuitively attractive but is challenging both in principle and in practice. It is generally agreed that the determination of best interests in a critically ill newborn baby involves the clinician in a difficult task of balancing and weighing a range of clinical factors whilst taking into account the opinions and attitudes of parents. A common and helpful approach to determining best interests is to attempt to weigh and balance the benefits of invasive life-support treatment with the burdens and risks of the treatment [4]. This ‘balance-sheet’ approach recognises that all medical treatments carry potential benefits to the patient but that they also necessarily incur burdens and risks. The essence of ethical practice is to ensure that the potential benefits of all treatments offered exceed the likely burdens and risks. Any proposed treatment should be assessed within this framework, considering a range of factors—physical, emotional, and social—and both short-term and long-term outcomes. The balance-sheet approach provides a helpful framework for ensuring that all aspects of a treatment decision are highlighted and incorporated into the decision-making process [5,6].

There are obvious challenges and difficulties in employing this balance-sheet approach to making life and death treatment decisions concerning a baby born at the limits of viability. The potential benefits of commencing invasive intensive care are enormous. Many children born at 22 weeks and 23 weeks who receive a prolonged period of intensive care will survive into adulthood. Data obtained in the UK from 2016 to 2019 indicate that of all babies born alive who received active care, approximately 35% at 22 weeks and 38% at 23 weeks achieved long-term survival [4]. Of those who survive at 22 weeks and 23 weeks, approximately 25% to 35% will have severe neurodevelopmental impairment, implying that at least two-thirds of survivors will not have severe impairment [7]. On the opposite side of the balance sheet, there are all the negative consequences of a prolonged period of neonatal intensive care, both for the infant concerned and for the parents, siblings and hospital staff.

Every experienced neonatologist is haunted by the experience of providing prolonged periods of intensive care in an extremely preterm infant who ultimately died after weeks or months of unpleasant and invasive treatment. It is all too possible for highly advanced and invasive medical treatment to become excessively burdensome and destructive; it can sometimes feel like a sophisticated kind of child abuse. Hence, clinicians can find themselves conflicted between a desire to offer a chance of life to every child who might survive and compassionate concern about the prolonged pain, distress and damage that futile medical treatment can result in.

The balancing of the benefits and burdens of invasive treatment for a child with a life-threatening illness is complex and difficult at any age. It is especially challenging, however, for neonatologists because they often find themselves caring for a child at a time when the end of his or her life more or less coincides with its beginning. When the end of life is with only within hours or days of birth, there are multiple uncertainties that make it difficult to be sure of the grounds on which compassionate decisions can be made.

When confronted with an extremely preterm infant born at the borders of viability, neonatologists find themselves having to judge and advise parents on what will offer more benefit than harm, without being able to know for certain what those benefits and harms will be. In effect, they find themselves expected to evaluate the potential future of a child based largely on statistical evidence of chances of survival and brain injury, as well as any evidence of damage or disease that can be identified in the present moment. Since the neonatologist cannot be certain what the future impact of interventions will be, she cannot be completely confident that her decisions will, on balance, be in the child’s interest. That is an uncomfortable position for a paediatrician who is committed to compassionate care to find him or herself in.

Paediatricians’ moral distress can hypothetically be relieved by some consequentialist bioethical theories that suggest the infant’s value can be wholly expressed as a function of the likelihood that she will recover at all, how closely her abilities approximate to what is considered normal and how much her parents want to keep her alive [8]. On those grounds, the infant’s life may be perceived as of instrumental value only; it has no value in itself and no moral distress need attach to withholding or withdrawing interventions that might prolong it. In effect, the neonatologist’s decisions would no longer need to represent a compassionate response to the infant as a person but rather a rational reaction to the infant’s perceived value (or lack thereof) in the present moment. Abandoning compassion in favour of logic as a motivator for correct moral action relieves the paediatrician of a moral dilemma, but for most paediatricians, it does not ‘ring true’. In practice, most neonatologists recognise the need to act with compassion to the unique infant that they are caring for.

Acknowledging that each extremely preterm infant has an inherent and absolute value does not, however, require the neonatologist to conclude that the infant’s life must always be prolonged at any cost. On the contrary, an informed analysis of the infant’s best interests is not at odds with ascribing inherent value to a living infant but rather underpins it. Compassionate care, whether at the end of life or at its beginning, demands that both doctor and parents always act in the child’s interests. There are circumstances under which the compassionate course of action is to rescue a child from interventions that are likely to be unhelpful or positively harmful. Withdrawing or withholding those interventions under such circumstances affirms, rather than denying, the inherent and absolute value of an infant. The same concern for the well-being of a child and family that, under other circumstances, is expressed by choosing to intervene to delay death can, when death is thought to be inevitable, be expressed by choosing not to intervene. Withholding or withdrawing an intervention that is not in the best interests of the individual infant is, in effect, an expression of wise and genuine compassion.

The decision to withdraw or withhold intensive treatment does not mean that we have accepted that the intrinsic value of a baby’s life is reduced, nor do we believe that we are justified in withdrawing life-support treatment because we believe that a baby’s life ‘is not worth living’. As physicians, we are in no position to make a judgement on the value of another person’s life. However, as medical professionals, we can and must make judgements about the value of medical treatments. We cannot determine whether or not a life is worth living, but we must decide whether or not a treatment is worth giving [9].

## 3. Counselling Parents

Wherever possible, detailed discussion and counselling with parents should commence before the birth of an infant at the borders of viability. In this situation, most parents are extremely distressed and concerned about the possible implications of death or severe disability for their precious child, and gentleness, sensitivity and expressions of empathy are absolutely vital. It is all too common for professionals to present statistics about survival and long-term impairment in an overly negative and insensitive way. In 2014, a group of parents of children born extremely preterm published an extraordinarily helpful paper in *Acta Paediatrica* [10]. Entitled *Our child is not just a gestational age. A first-hand account of what parents want and need to know before premature birth*, the article should be required reading for all neonatologists. The authors emphasise the need for neonatologists to adopt a personalised approach and to listen carefully to individual parents’ questions and concerns rather than merely repeating impersonal population statistics.

“We need to trust you. Do not tell us that babies at 22 and 23 weeks do not survive. Do not tell us that most preterm babies are disabled...Do not tell parents that their child will have a negative impact on their family. You do not have data to support these claims…Our discussions should be about values and not so much about percentages.” “Words are important. Before our child’s birth, do not ask us if we want to do ‘everything’ or ‘nothing’. Have you ever met parents who wanted ‘nothing’ for their children?”. “Do not take away the hope we have. There is always hope that we will deliver tomorrow. There is hope that we will be able to spend some time with our child. There is hope that we will be able to spend some time with our child. There is hope that we can survive the death of our child with positive memories. Do not abandon us. Instead tell us that you will be there whatever happens” [10].

If it is agreed with the parents that no active intervention will be commenced following the delivery of their baby, it should be explained that palliative or ‘comfort-focussed’ care will be commenced immediately, and the nature and goals of palliative care (see below) should be explained. It is important that a senior neonatologist is present at the delivery in order to rapidly assess the condition of the baby and confirm that the findings are consistent with what had been anticipated.

Despite the sophistication of contemporary antenatal investigations, sometimes the condition of the baby at birth is unexpectedly good. The medical staff should discuss with the parents whether the estimated gestation and prognosis were accurate and whether the planned palliative approach is still appropriate. Active stabilisation and resuscitation should not be delayed if they are thought to be in the baby’s best interests [7]. On the other hand, if active neonatal management has been agreed before birth but the baby is born in unexpectedly poor condition, it is the responsibility of the most senior neonatal professional present to decide whether ongoing attempts at stabilisation and resuscitation are in the baby’s best interests. If not, this should be communicated sympathetically but unambiguously to parents, and withdrawal of resuscitation efforts and commencement of palliative care should be actively recommended.

## 4. Goals and Philosophy of Palliative Care

Clarity about goals is essential when palliative care is being contemplated, yet most practising neonatal staff do not spend much time reflecting on the philosophy and fundamental aims of neonatal intensive care. Conceptually, the goals of routine neonatal medical care and palliative care are quite different, and the two modes of caring must be clearly distinguished.

The goals and priorities of ‘routine’ or ‘standard’ neonatal care can be summarised as follows. First, the overriding priority is to preserve and prolong survival. The second priority is to reverse or treat underlying pathological processes and to support fundamental physiological functioning and homoeostasis (respiration, circulation, metabolism, etc.). Third, the goal is to minimise pain and distress during neonatal care and to minimise the adverse effects of care on the developing nervous system. Fourth, the goal is to provide accurate information and psychological support for parents and family. The routine clinical management policies for the NICU should represent this hierarchy of priorities.

In contrast, the goals and priorities of palliative care are very different [11]. Where survival is no longer considered possible, the over-riding priority is to prevent or minimise pain and discomfort and to treat any distressing symptoms. That includes symptoms that are primarily physical, such as dyspnoea, recurrent convulsions, vomiting and so on. However, it also includes reducing distress that is not physical but rather emotional or psychological. A priority is to maximise the opportunity for private, loving interaction between the dying baby and his or her parents and the wider family. Physical contact and tender loving care are of central importance for the dying baby, and if the parents are not able to provide this basic care (for whatever reason), then it is the duty of the neonatal staff to act as substitute parents. Palliative care for the infant extends to offering psychological support for her parents and family as they go through the profound and painful life experience of accompanying their baby to death and beyond. Clinical experience shows how difficult it is to predict how long a baby with a particular combination of clinical features may survive without intensive care. Whether the process of dying lasts minutes, hours, days or months, the duty of the health team is to accompany the baby and the parents along the journey and to reassure the parents that we will never abandon them [12].

It is the infant herself, however, who is the primary focus of palliative care in neonates. The newborn infant, even if she has normal cognitive ability, has little prior experience of existence and, as far as we know, lacks rational appreciation of a future that might or might not happen. That does not, however, mean that she is incapable of suffering in the present moment. There is considerable evidence that infants experience pain in broadly the same way that adults do; that is, in emotional, existential or spiritual dimensions, as well as the physical dimension [13,14]. We do not know if that is true of other symptoms in the neonatal period, such as dyspnoea or cerebral irritation, but it seems wise to assume that it is. The association of anxiety with dyspnoea or disordered cognition, for example, is fundamental to human existence; it seems probable that being unable to breathe or to think clearly is as frightening to humans while they are neonates as when they are older children or adults. Compassion for our patients means that we will spare no efforts to manage distressing symptoms to the best of our abilities. Although its application to neonates is currently often based only on extrapolation and theory, the therapeutic science that underpins effective symptom control more generally is well supported by empirical evidence. What follows is not an attempt to offer definitive guidance in symptom management or to review the science on which such guidance is based (the interested reader is referred to larger reference sources, such as the *Oxford Textbook of Palliative Care in Children* [15], and the *Oxford Textbook of Palliative Medicine* [16]). Our aim is to illustrate the way in which good control of symptoms ensures that medical care of the neonate remains consistent with an ethic of compassion, even when it is no longer aimed at a cure.

Redirecting care from intensive to palliative usually means that there are some interventions that should be withdrawn. The philosophical framework of palliative care makes clear that the aim of palliative medicine intervention is neither to prolong nor to shorten life. Death should not be hastened, for example, by withdrawal of hydration or nutrition with milk feeds. However, dying babies do not usually require large amounts of fluid or milk. A preterm baby in a terminal phase frequently requires only 40–60 mL/kg/day (or even less), usually administered by a nasogastric or orogastric tube. There are nevertheless circumstances under which effective palliative care might means discontinuing feeds or (more rarely) nutrition. If there is complete intestinal obstruction, for example, there is no chance that fluids can be absorbed. As death becomes imminent, the benefits of fluid and/or nutrition in any individual child may become less clear and, since they can be associated with some harms, including uncomfortable blood tests, it might sometimes become appropriate to discontinue them. It remains important under those circumstances to ensure good mouth care, and it may be appropriate to give the baby something to suck on. It is very important that the fluid and nutrition management policy is discussed with parents and that they are reassured that the staff are not deliberately starving or dehydrating their child.

Some forms of monitoring also become inappropriate when there is no longer any prospect of prolonging a child’s life. Routine blood tests and unnecessary investigations and monitoring should be rigorously avoided. Because the goal of care is not to prevent further injury or to normalise physiology, there is usually no need to give oxygen or to monitor physiological variables, such as oxygen saturation or serum glucose and electrolytes. At the same time, in order to use symptom-control medications effectively, it is important to individualise therapy according to a baby’s response and changing condition. Clinical monitoring or intervention are only indicated where they are needed to minimise distress; therefore, the baby continues to need to be observed carefully.

Maintaining intravenous access can be invasive and is often inappropriate in neonatal palliative care, especially if the family prefers to care for their child at home or in hospice. Most of the medications needed in neonatal palliative care can be given by other routes. The subcutaneous route is reliable and avoids multiple attempts at venous cannulation, although it can become painful if a needle or cannula is left in situ for too long. Benzodiazepines and some opioids are well absorbed by transmucosal routes (buccal, rectal, nasal and even transdermal). The palliative care team or pharmacist will be able to advise on the most appropriate way to deliver symptom-control medications.

‘Palliative care’ is sometimes wrongly thought to be synonymous with ‘withdrawal of care’. From a humanitarian perspective, we may withdraw treatment, but we never stop providing care. In addition to discontinuing some interventions, effective palliative care usually consists of introducing others. Many babies require some form of analgesia; pain is not an inevitable accompaniment to dying in the neonatal period, but it is common. If simple analgesia, such as paracetamol or non-steroidal anti-inflammatory drugs, are inadequate, opioids are the mainstay of pain management in the neonatal period, as at other times. The correct dose is the dose that is effective while carrying the least risk of adverse effects. The dose will depend on the child’s weight, the clinical evidence of the severity of his or her pain and distress and whether or not she has been exposed to opioids before. Usual practice is to select a starting dose on the basis of weight alone to be given ‘as needed’ in response to behaviours that the care team and parents feel demonstrate pain [17]. After 48 h or so, it will be clear how many of those ‘as-needed’ doses are required. If the child needs only one or two doses a day, it is reasonable to continue them ‘as needed’. If she needs more than that, then in addition, she should have a regular ‘background’ dose over 24 h in order to avoid her having to experience pain as much as possible. Each unit will have its own guidelines for prescribing ‘background’ opioids, and there are also palliative care resources available. It is important to prescribe ‘as needed’ (breakthrough) whenever regular (background) opioids are started, and the dose of breakthrough treatment should always be increased in line with increments in the background dose so that the two remain in the same proportion.

Dyspnoea is a subjective sensation that breathing has become uncomfortable. Observable changes in the work or rate of breathing are often, though not always, associated with dyspnoea. The association with abnormal blood gases is less clear, and neither low oxygen saturation nor hypercarbia on its own necessarily indicates discomfort. Reassuring physical contact, especially cuddles from parents, are highly effective in relieving dyspnoea and anxiety (see below). Blowing air on the face can relieve the sensation of dyspnoea through activation of receptors in the skin [18]. Pharmacological measures include opioids and benzodiazepines, especially midazolam, both of which act directly on the respiratory centre.

Benzodiazepines have an additional benefit in relieving anxiety. The dose of benzodiazepines and opioids required to relieve dyspnoea is substantially lower than that which will cause apnoea. It is important to reassure parents and staff accordingly; otherwise, they may worry that treating dyspnoea will lead to the hastening of the child’s death. It is important to note that paralysing muscle relaxants have no place in managing dyspnoea or in palliative care generally. They do not reduce the patient’s distress but exacerbate it by inducing a feeling of suffocation.

Cerebral irritation is a form of pain that results from acute brain tissue injury, especially hypoxic–ischaemic injury, although it may also be associated with central nervous system infections and metabolic disorders. We do not know exactly what a newborn experiences, but it seems reasonable to assume that the combination of inflammation, neuroexcitation and disordered cognition leads to a complex pain state that includes headache, confusion and anxiety [19]. Alongside adequate analgesia and physical contact, as mentioned above (if possible), the mainstay of medical management is phenobarbital, gabapentin or other sedative anticonvulsants to reduce neuroexcitation, as well as benzodiazepines to manage anxiety.

While there are some helpful resources to support neonatal staff in offering palliative care [12], it is recommended that if there are complex symptoms, advice should be sought from the specialist paediatric palliative care team if one is available locally or in a national centre.

## 5. ‘Total Pain’

The pioneers of adult palliative medicine described the concept of ‘total pain’ [20]. This is pain that encompasses every aspect of the individual—physical, cognitive, affective, relational, social, spiritual or existential. In palliative care of the newborn, the idea of ‘total pain’ needs to be conceived not only in the infant but also in her parents. In addition to the baby’s own pain and distress, there are aspects of ‘total pain’ that are suffered by the parents. The challenge for health professionals is to address every aspect of this total pain. In contrast to the narrow technical skills that much of modern neonatology requires, palliative care requires a broad understanding of many different aspects of human experience and a sensitivity to the unique constellation of issues that every patient and every family bring.

## 6. Expert–Expert Relationships

When discussing treatment and care options with parents in the antenatal and neonatal periods, the approach must be collaborative rather than paternalistic or impersonal. The model of an “expert–expert” relationship with parents can be helpful [21]. The neonatologist has expertise in the diagnosis, prognosis and treatment options available to a newborn with a life-threatening illness or malformation. However, the neonatologist must bring not only technical expertise to the discussion but also the essential virtues of the physician: compassion, empathy, wisdom, humanity and moral integrity. On the other hand, parents have expertise too—in their family background, life history, values, philosophical beliefs and so on. The basis of expert–expert relationships must be one of mutual respect. The neonatologist can expect parents to respect their professional expertise, but she too must respect the particular expertise of the parents. Together, we try to find agreement on the way forward, a consensual decision that meets the concerns of both parties. This quest for consensus requires honesty, openness and transparency.

Of course, the above description is idealised, and in reality, this collaborative approach can be demanding and time-consuming. Some parents find sharing the burden of responsibility for decision making extremely painful and distressing, and excellent communication and listening skills are essential.

## 7. Recognising and Celebrating the Unique Identity of Each Child

For parents, nothing can take away the opportunity to meet their unique child face to face. Even if their baby only lives for a matter of minutes, hours or days, the child has an identity and a name. It is vitally important that hospital staff and the routines of neonatal care units do not impede the freedom of parents and close family and friends to spend time in private with their child. Wherever possible, the baby should be held and cuddled, not just observed. Those precious minutes and hours meeting their child provide experiences that are often vividly relived and remembered for the rest of their lives. Photographs are taken, and mementos, such as footprints or an identity bracelet, are kept. Siblings and the extended family are able to meet and cuddle the child. Where appropriate, religious or other ceremonies can be arranged. All of this means that the child becomes a social reality, a never-forgotten member of the community.

Here is an attempt to summarise the intuitions of many parents whose babies we have cared for as paediatricians. “My baby is a unique individual with a history, an identity and a name. My baby is a person, not a thing; a ‘he’ or ‘she’ not an ‘it’. My baby must be treated with gentleness and respect by those who are caring for her. My baby can never be replaced although I may have other babies in the future. If my baby’s outlook is hopeless, then it may be the most compassionate act to withdraw treatment and ‘keep her comfortable’ knowing that she will die. Withdrawing treatment and allowing my baby to die, is not the same as deliberately killing her. If my baby does die, I need permanent reminders and mementoes of her short existence, to honour her memory as a unique person, my beloved and never-forgotten child”.

## 8. Maintaining Continuity of Care

Wherever possible, one named consultant neonatologist should provide a clear lead in terms of management policy and communication with parents and should ensure that all conversations and the evolving management plan are well documented in the medical records. Regular discussion with the parents should include how the baby’s condition is evolving, what the implications are for how long the baby may live and what are the options in terms of pain relief and symptom control. Parents often have valuable perceptions as to whether their baby is suffering distress, and their concerns and anxieties should be listened to and respected. It is important to try to ensure continuity of care over nights and weekends, when staff changes may cause disruption and anxiety. In particular, it is vitally important to avoid any psychological sense of ‘abandonment’.

## 9. Emotional Reactions to Care of the Dying Baby

When confronted by suffering and impending death, there is a natural human reaction to withdraw and turn away. It is much more pleasant to concentrate our time and attention on the babies who are getting better and to spend time with their parents. However, in palliative care, we must overcome these natural human reactions. Instead, our first duty is to ‘be there’ with the parents and their dying baby. We need to reassure them that whatever happens, we will never abandon them; we will always be there for them; we will accompany them on this strange journey, whatever happens, however long it takes.

This is why the symbolism of the neonatologist spending time in the room with parents and their baby is so significant. Gently examining the child, looking at the observation charts, taking time to speak to the parents or just to put a hand on a shoulder can speak volumes. Sometimes just to sit in silence with the parents can be immensely helpful and reassuring. At one level, we may feel powerless; we can do nothing for the baby from a technical medical sense. However, the symbolism of our concern, engagement and compassion is of enormous significance.

One of the essential characteristics that neonatal staff should display to the dying baby is *respect*—respect for the essential dignity of the human person, however malformed and distorted by the consequences of disease and prematurity. Parents are very sensitive to the hidden attitudes of staff, and if they sense an attitude of contempt or indifference to their child, this can be very hurtful. We communicate an attitude of respect by spending time with the baby, by the way we touch and handle the baby and parents, by our words and by our actions. Staff may feel that in this distressing situation, they should always maintain a professional façade and act in a cool and unemotional way. However, in reality, parents often find it helpful if staff verbalise their own feelings of human emotion and distress.

## 10. Supporting Staff through Palliative Care and Neonatal Death

The senior medical and nursing staff on the neonatal unit have a vital role in providing psychological and professional support for junior staff involved in palliative care. It is not unusual for junior staff to feel abandoned by their senior colleagues at a time when they feel inexperienced, disempowered and out of control. It is essential that the named consultant is seen to be closely involved not only talking to the parents but also consulting with the junior neonatal staff and explaining the reasoning behind the management plan. If the period of palliative care is protracted, it is very helpful to hold informal private meetings for staff to discuss and express their feelings openly. Similarly, once death has occurred, a meeting for debriefing and discussion is often helpful. Staff may require reassurance that their actions and decisions were appropriate and, because neonatal death is relatively rare, these informal meetings provide an excellent opportunity for education and discussion on the philosophy and practicalities of palliative care.

Burnout and depression are relatively common in staff who are overexposed to neonatal death, and there is increasing recognition of the need for some form of regular individual supervision or counselling for frontline staff who are closely involved in counselling and supporting parents [22]. It may be appropriate to encourage staff to rotate to less stressful areas for a time, such as outpatient duties or clinical research, when the pressures of palliative care become overwhelming. We must always remember that we have a duty of care to our staff as well as to our patients.

## 11. Conclusions

Although palliative medicine is now a paediatric specialty in itself, the philosophy on which it is based is relevant to any team having to care for children at the end of life. Providing skilled palliative care for dying babies is an essential part of modern neonatal medicine and nursing. At present, many neonatal staff remain ambivalent and uncertain about this aspect of neonatal care, and it is sadly true that many babies appear to die with inadequate and unskilled care. There is still a need to transform attitudes within the profession. To ensure that a baby dies at peace, pain-free and surrounded by her supported family should be seen not as failure but as a triumph of modern neonatal care. 
